# Development of A New Mouse Model for Intrahepatic Cholangiocellular Carcinoma: Accelerating Functions of Pecam-1

**DOI:** 10.3390/cancers11081045

**Published:** 2019-07-24

**Authors:** Ihtzaz Ahmed Malik, Gesa Malik, Philipp Ströbel, Jörg Wilting

**Affiliations:** 1 Department of Anatomy and Cell Biology, University Medical Center Goettingen, Kreuzbergring 36, D-37075 Goettingen, Germany; 2 Institute of Pathology, University Medical Center Goettingen, Robert-Koch-Strasse 40, D-37075 Goettingen, Germany

**Keywords:** liver cirrhosis, inflammation, chemokines, bile-duct-cancer, proliferation, cholangiogenesis

## Abstract

Due to the lack of suitable in-vivo models, the etiology of intrahepatic cholangiocellular carcinoma (ICC) is poorly understood. We previously showed the involvement of platelet endothelial cell adhesion molecule-1 (Pecam-1/CD31) in acute liver damage. Here, we developed a model of ICC using thioacetamide (TAA) in drinking water of wild-type (WT)-mice and Pecam-1-knock-out (KO)-mice. Gross inspection and microscopy revealed liver-cirrhosis and ICC in both groups after 22 weeks of TAA. The severity of cirrhosis and ICC (Ck-19-positive) was reduced in Pecam-1 KO mice (stage-4 cirrhosis in WT vs. stage-3 in KO mice). Tumor networks (accompanied by neutrophils) were predominantly located in portal areas, with signs of epithelial-to-mesenchymal transition (EMT). In serum, TAA induced an increase in hepatic damage markers, with lower levels in Pecam-1 null mice. With qPCR of liver, elevated expression of Pecam-1 mRNA was noted in WT mice, in addition to Icam-1, EpCam, cytokines, cMyc, and Mmp2. Thereby, levels of EpCAM, cytokines, cMyc, and Mmp2 were significantly lower in Pecam-1 null mice. Lipocalin-2 and Ccl5 were elevated significantly in both WT and Pecam-1 null mice after TAA administration. Also, EMT marker Wnt5a (not Twist-1) was increased in both groups after TAA. We present a highly reproducible mouse model for ICC and show protective effects of Pecam-1 deficiency.

## 1. Introduction

Hepatocellular carcinoma (HCC) and intrahepatic cholangiocellular carcinoma (ICC) are the most common primary hepatic malignancies, accounting for roughly 70% and 15% of cases, respectively [1,2]. Despite the large number of ICC in primary liver cancer, studies on the disease are very scarce. Treatment depends on the site and extent of the lesion, and only surgical resection can improve the outcome significantly. However, due to large tumor size, surgery is not applicable for the majority of cases. Even with modern disease management, there has still not been any significant improvement in the patients’ prognosis; for example, in the United States of America, the 5-year survival rate for patients with ICC has remained at a low 8% (American Cancer Society, 2018, [3]). Unfortunately, the lesions are usually diagnosed only at advanced stages, and there are no drugs specifically approved for the treatment of ICC so far.

ICC is an epithelial malignancy emerging from transformed cholangiocytes [4,5,6]. The risk factors for ICC are not well understood so far. However, there is growing evidence that activation of inflammatory pathways, which disturb the hepatobiliary transport, are part of the etiology. This influences the growth and apoptotic behavior of biliary cells and induces pro-oncogenic cascades, such as the release of cytokines (e.g., IL-6, IL-8) and upregulation of growth factors (e.g., hepatocyte growth factors, HGF) [4,5,6,7]. A link between inflammation and cancer was proposed quite some time ago. Thereby, migration of leukocytes is a hallmark of inflammation, which is controlled by cytokines, chemokines [8] and adhesion molecules [9].

Platelet endothelial cell adhesion molecule-1 (PECAM-1/CD31) is a homo- and heterophilic cell adhesion molecule of 130-kDa expressed on the surface of endothelial cells and leukocytes [10,11,12]. PECAM-1 has important though not fully understood roles in leukocyte trans-endothelial migration (TEM) during immune surveillance and inflammation. Thereby, it facilitates leukocyte transmigration by heterophilic interactions via its extracellular domain [12,13]. PECAM-1 is also known to play a role in fibrosis and spreading of primary tumors by controlling processes such as vascular permeability and capillary morphogenesis [14]. High PECAM-1 expression has been found in human brain gliomas [15], carcinoma of the cervix [16], renal cell carcinoma [17], lung cancer [18] and breast cancer [19]. PECAM-1 has metastasis- and proliferation-promoting effects, which seem to be independent of tumor type, but have most clearly been shown in studies on late-stage melanoma progression [14]. In line with this, inhibition of PECAM-1 revealed a decrease in proliferation of murine melanoma and mammary carcinoma cells [20]. In vivo, the positive effects of PECAM-1 inhibition on tumors, such as reduction in lung metastases, have been noted in PECAM-1-null mice, or after inhibition of Pecam-1 with specific antibodies [14,20]. However, a potential role of PECAM-1 in ICC has not been investigated so far.

Rodents have regularly been used in the past as preclinical models for the study of liver cancer. However, the majority of studies have focused on HCC [21]. The induction of ICC, especially in the genetically highly versatile mouse model, has remained enigmatic. Thioacetamide (TAA) is an organosulfur compound and well-known hepatotoxin [22]. A single injection of TAA evokes reversible damage of the liver parenchyma in rodents [23]. We have previously shown that Pecam-1-regulated pathways play an important role during tissue damage, and we could further demonstrate liver-protective effects of PECAM-1 by using Pecam-1-deficient mice in acute inflammation models [24,25,26]. Thereby, PECAM-1 appeared to be an interesting candidate for inflammation-triggered liver cancer. Here we sought to develop a preclinical mouse model for the study of primary ICC by continuous application of TAA. We used oral administration of TAA over a long period in both wild-type (WT) and Pecam-1 null mice. We present the first mouse model for ICC and we show that the loss of Pecam-1 has protective effects. Accordingly, the use of anti-PECAM-1 therapeutics might provide a strategy for the treatment of hepatic inflammatory disorders and their sequelae.

## 2. Results

### 2.1. Gross Anatomy and Microscopy of Liver in WT- and Pecam-1-KO-Mice after TAA-Administration

**Figure 1:**
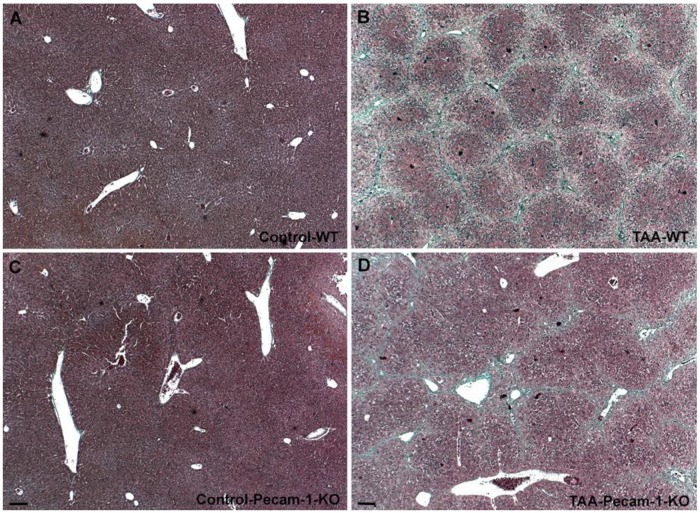
Trichrome staining of livers from WT mice (**A**,**B**) and Pecam-1 KO mice (**C**,**D**). Livers of Control WT (**A**) and KO (**C**) mice, which received only water. (**B**,**D**) After 22 weeks of TAA, cirrhosis is visible, and is more severe in WT mice. Scale bar = 100 µm.

**Figure 2:**
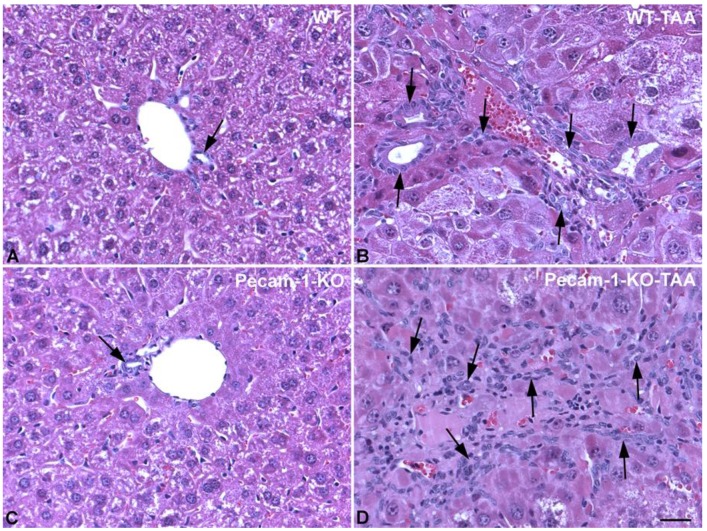
HE staining of livers from WT mice (**A**,**B**) and Pecam-1 KO mice (**C**,**D**). Control WT (**A**) and KO (**C**) mice, which received only water and show normal liver with regular bile ducts (arrow). (**B**,**D**) After 22 weeks of TAA, dilated bile ducts and networks of bile ducts are visible (arrows). Scale bar = 35 µm.

**Figure 3:**
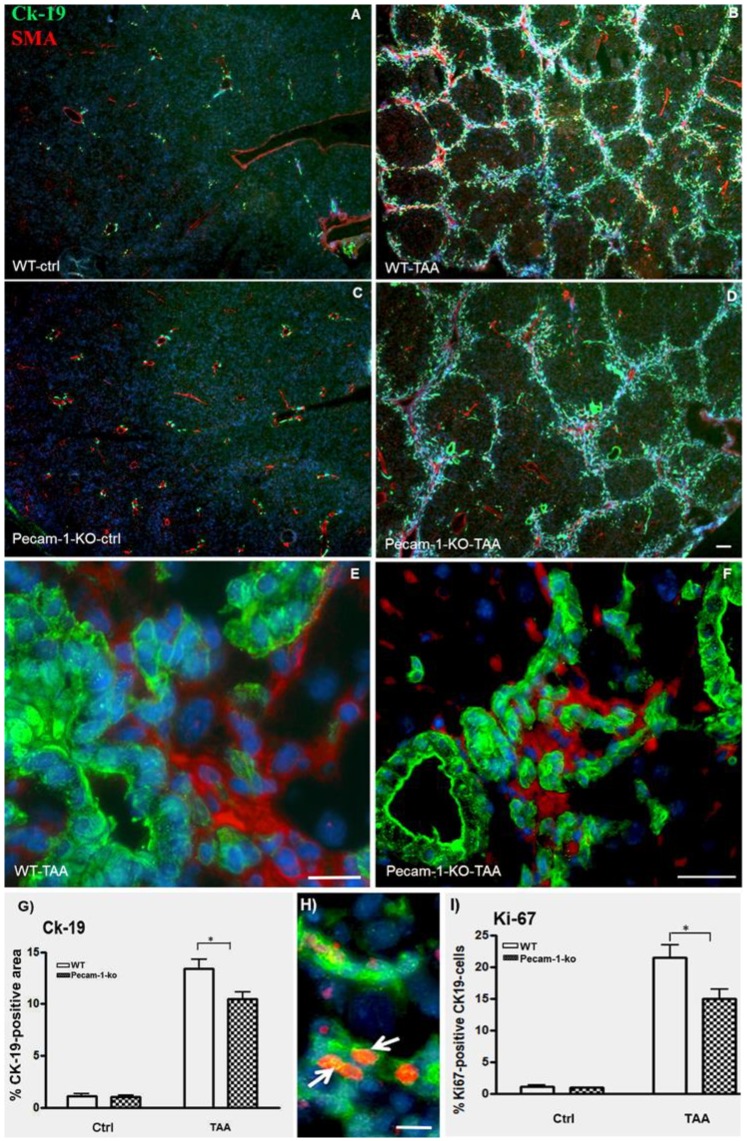
Detection of Ck-19 (green, marker for biliary cells) and α-SMA (red, marker for myofibroblasts and SMCs) by double immunofluorescence staining in WT (**A**,**B**,**E**) and Pecam-1 KO mice (**C**,**D**,**F**). Control livers of WT (**A**) and Pecam-1 KO mice (**C**), which received pure water and show a regular pattern of bile ducts and larger vessels. After 22 weeks of TAA to WT (B) and Pecam-1 KO mice (**D**), hyperplasia of Ck-19-positive cells is evident. (**A**–**D**) Original magnification 2.5× objective. Scale bar = 50 µm Higher magnification shows that Ck-19-positive bile duct cells are αSMA-negative in WT (**E**) and Pecam-1 KO mice (**F**). Scale bar = 25 µm in (**E**) and 35 µm in (**F**). Nuclei were counter-stained with DAPI (blue). (**G**) Quantification of Ck-19-positive cells in WT and Pecam-1 KO mice using ImageJ software. Note the highly significant increase of Ck-19 after TAA in both groups, and the significantly (*) higher value in WT vs. KO mice. (**H**) Detection of Ck-19 (green) and Ki-67 (red, cell proliferation marker) by double immunostaining. A typical example of WT mouse is shown. After 22 weeks of TAA, hyperplasia of Ck-19-positive cells is evident. Ki-67^+^ ICC are marked with white arrows. Nuclei were counter-stained with DAPI (blue). Original magnification 20× objective. Scale bar =15 µm. (**I**) Quantification of Ki-67-positive Ck-19 cells in WT and Pecam-1 KO mice. Note the highly significant increase of Ki-67 after TAA in both groups, and the significantly (*) higher value in WT vs. KO mice.

At macroscopic inspection, the livers of the TAA-treated animals revealed clear signs of cirrhosis and cancer. The livers were reduced in size and their surface showed the typical nodular appearance, in contrast to the smooth surface in control animals. The incidence of cirrhosis and ICC reached 100% in both WT- and Pecam-1 KO mice after 22 weeks of TAA-administration, as evidenced by trichrome and HE staining (Figure 1 and Figure 2). Histopathology of the livers presented an adenocarcinoma in conjunction with extensive desmoplasia, proliferating ducts and abnormal luminal profiles (Figure 1, Figure 2 and Figure 3).

Moreover, the inflammation grade of the livers and staging of cirrhosis were diagnosed by an experienced pathologist. Whereas WT-mice generally presented with stage 4 liver cirrhosis, all Pecam-1 KO mice had stage 3 cirrhosis. Similarly, with just one exception, the grade of inflammation of WT mice was slightly higher than that of Pecam-1 KO mice (Table 1).

### 2.2. Immunohistological Identification of ICC in Murine Liver after TAA-Administration

Double immunofluorescence staining of cytokeratin (Ck)-19 and α-smooth muscle actin (αSMA) of normal liver revealed positivity for biliary cells and smooth muscle cells (SMCs), respectively, in normal liver of both WT and Pecam-1 null mice (Figure 3A,C). After TAA administration, the number of Ck-19-positive cells increased mainly in the interlobular septa (Figure 3B,D). αSMA expression was detected in SMCs of interlobular hepatic arteries as well as portal and central veins, and additionally in myofibroblasts of the fibrotic septa. Notably, the Ck-19-positive bile duct cells were αSMA-negative (Figure 3E,F). The number of Ck-19-positive cells was significantly higher in WT-mice than in Pecam-1 KO mice (Figure 3G).

Hyperplasia of CK-19-positive cells was confirmed by double staining with the proliferation marker Ki67. Notably, we observed a significantly higher percentage of Ki67-positive cells in WT mice as compared to Pecam-1 KO mice after 22 weeks of TAA treatment (Figure 3H,I).

**Figure 4:**
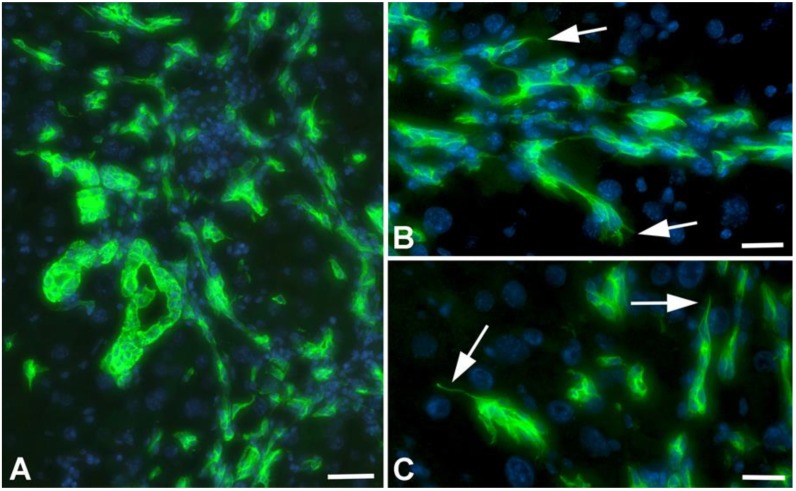
Detection of Ck-19 (green) in liver of TAA-treated WT-mice. (**A**) Overview showing dilated bile ducts, biliary networks and Ck-19+ scattered cells. Scale bar = 70 µm. (**B**,**C**) Higher magnification showing filopodial extensions (arrows) of Ck-19+ cells, indicative of tip cells. Scale bar = 40 µm.

Further studies of TAA-treated animals with anti-Ck-19 antibodies showed that besides dilated bile ducts and networks of biliary cells, there were numerous Ck-19-positive scattered cells, indicating epithelial-to-mesenchymal transition (EMT) (Figure 4A). Regularly we observed filopodial extensions of biliary cells, which is also indicative of EMT of tip cells during aberrant cholangiogenesis (Figure 4B,C).

**Figure 5:**
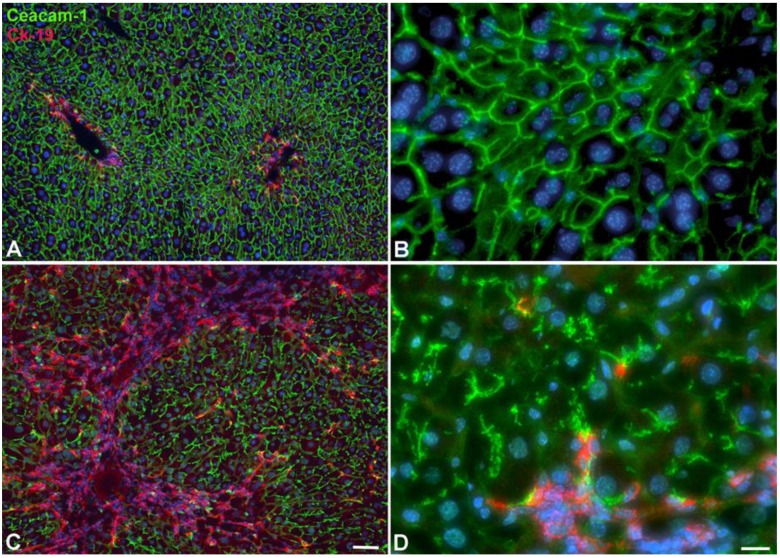
Development of ICC in WT-mice after 22W of TAA in drinking water. (**A**,**B**) Control animal. (**C**,**D**) TAA-treated animal. Anti-Ck-19 staining (red; bile duct marker) and anti-Ceacam-1 staining (green; bile canaliculi). Note development of ICC (red area in **C**), and the disruption of bile canaliculi in hepatocytes (**D**). Scale bar = 60 µm in (**A**,**C**); and 15 µm in (**B**,**D**).

We performed additional studies on the bile duct system with antibodies against carcinoembryonic antigen-related cell adhesion molecule 1 (Ceacam-1), a biliary glycoprotein, to demonstrate the bile canaliculi in hepatocytes. We observed disruption of bile canaliculi in parallel to the development of ICC (Figure 5). This suggests disruption of hepatocyte polarity and function in our model.

### 2.3. Infiltration of Neutrophilic Granulocytes in Liver after TAA-Administration

**Figure 6:**
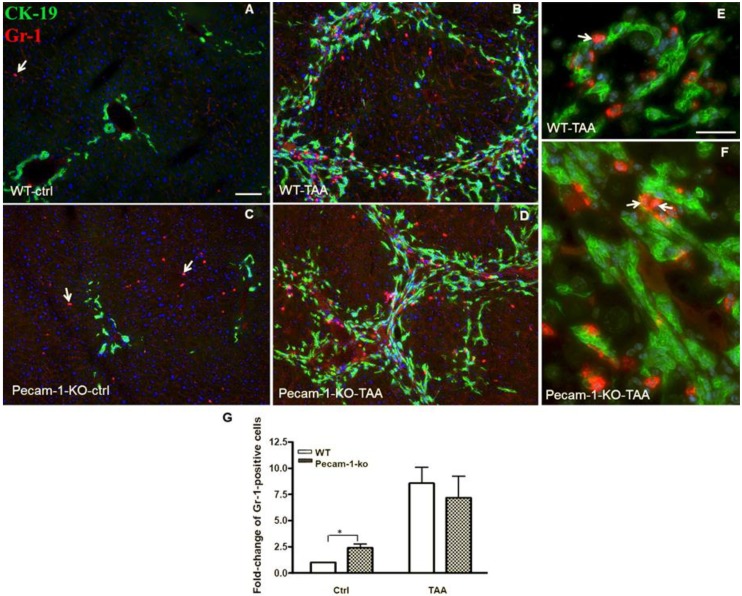
Detection of Ck-19 (green, biliary cells) and Gr-1 (red, marker for neutrophils) by double immunofluorescence staining in WT and Pecam-1 KO mice. Control WT-livers (**A**) contain significantly less neutrophils compared to the liver of Pecam-1 KO mice (**C**), which received only water. Their numbers are increased after 22 weeks of TAA administration in both WT (**B**) and Pecam-1 KO mice (**D**). Nuclei were counter-stained with DAPI (blue). Original magnification 10× objective. Scale bar = 200 µm in (A). (**E**,**F**) Higher magnification of WT (**E**) and Pecam-1 KO mice (**F**) after TAA-administration; 40× objective, Scale bar = 300 µm. White arrows indicate liver neutrophils. (**G**) Quantification of Gr-1-positive cells in WT and Pecam-1 KO mice. Note the massive increase after TAA, and the significantly higher number of Gr-1-positive cells in untreated KO vs. WT mice. Significance was accepted at * p ≤ 0.05.

Double immunofluorescence staining of Ck-19 and Gr-1 (marker of neutrophils) revealed a small number of positive cells in the livers of control WT mice. The quantification of Gr-1-positive cells demonstrated a significantly higher number in untreated (control) Pecam-1 KO mice compared to WT mice. The number of Gr-1-positive cells increased after TAA administration in both groups of mice, with no obvious quantitative differences between the groups. Of note, neutrophils were mainly found adjacent to Ck-19+ cells in tumor-containing, interlobular septa (Figure 6A–G).

### 2.4. Localization of Macrophages in Liver after TAA-Administration

**Figure 7:**
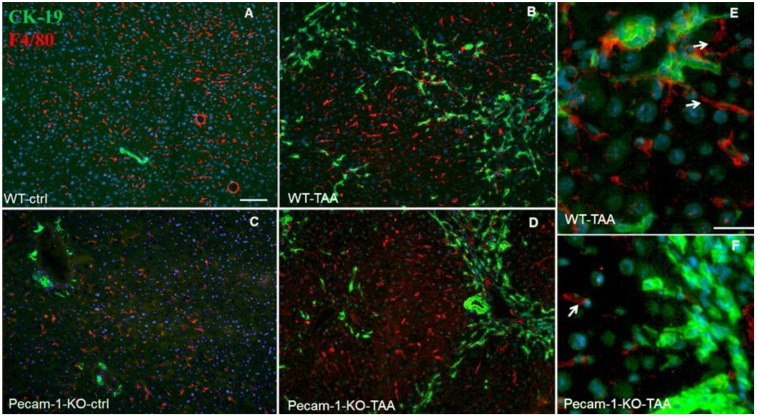
Detection of Ck-19 (green, biliary cells) and F4/80 (red, marker for macrophages) by double immunofluorescence staining in WT (**A**) and Pecam-1 KO control (ctrl) mice (**C**), which received only water, and after TAA-administration, nuclei were stained with DAPI (blue). In control mice (**A**,**C**), macrophages are regularly distributed. After TAA, macrophages are mainly found adjacent to hepatocytes, but some are also located in the vicinity of Ck-19+ cells (**B**,**D**–**F**). White arrows indicate liver macrophages. Scale bar = 300 µm in (**A**–**D**), and 25 µm in (**E**,**F**).

By means of Ck-19 and F4/80 immunostaining, it was possible to detect macrophages (F4/80-positive) along the sinusoids in normal liver. In contrast to neutrophils, in both TAA-treated WT and Pecam-1 KO mice we found macrophages predominantly in their normal intra-sinusoidal position in the hepatic nodules, and there was no accumulation in the ICC-containing septa, although macrophages were regularly present in these areas, too (Figure 7).

### 2.5. Elevated Liver Markers in Serum of WT and Pecam-1 KO Mice after TAA Administration

**Figure 8:**
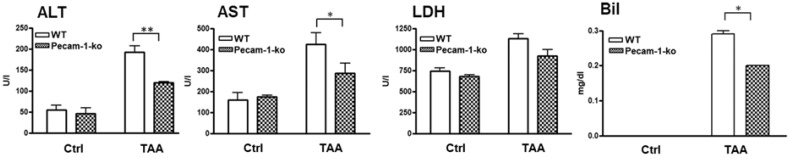
Studies on hepatic damage markers in serum. Alanine aminotransferase (ALT), aspartate aminotransferase (AST), lactate dehydrogenase (LDH) and bilirubin (Bil) concentrations in serum are shown. Note increase in liver damage by TAA, but significantly lower levels of ALT, AST and Bil in Pecam-1 KO mice after TAA treatment, as compared to TAA-treated WT mice. The results are compared with WT controls, which received only water. Results represent the mean ± SEM of 4–10 experiments. Significance was accepted at * p ≤ 0.05, ** *p* ≤ 0.01.

To examine liver damage, serum levels of alanine aminotransferase (ALT), aspartate aminotransferase (AST), lactate dehydrogenase (LDH), and bilirubin (Bil) were analyzed in WT and KO mice (Figure 8). The serum concentration of all studied markers was increased in both groups after TAA administration compared to WT controls. However, the values were significantly higher in WT than in Pecam-1 KO mice, with the exception of LDH. Thereby, ALT revealed a highly significant difference between WT (192 ± 15-U/L) and KO mice (119 ± 3-U/L). A significant difference between the two groups of mice was also noted for AST (WT: 424.5 ± 55-U/L vs. KO: 288 ± 46-U/L) and Bil (WT: 0.29 ± 0.1 mg/dL vs. KO: 0.2 ± 0-mg/dL) (Figure 8).

### 2.6. Changes in Hepatic Gene Expression of Adhesion Molecules

**Figure 9:**
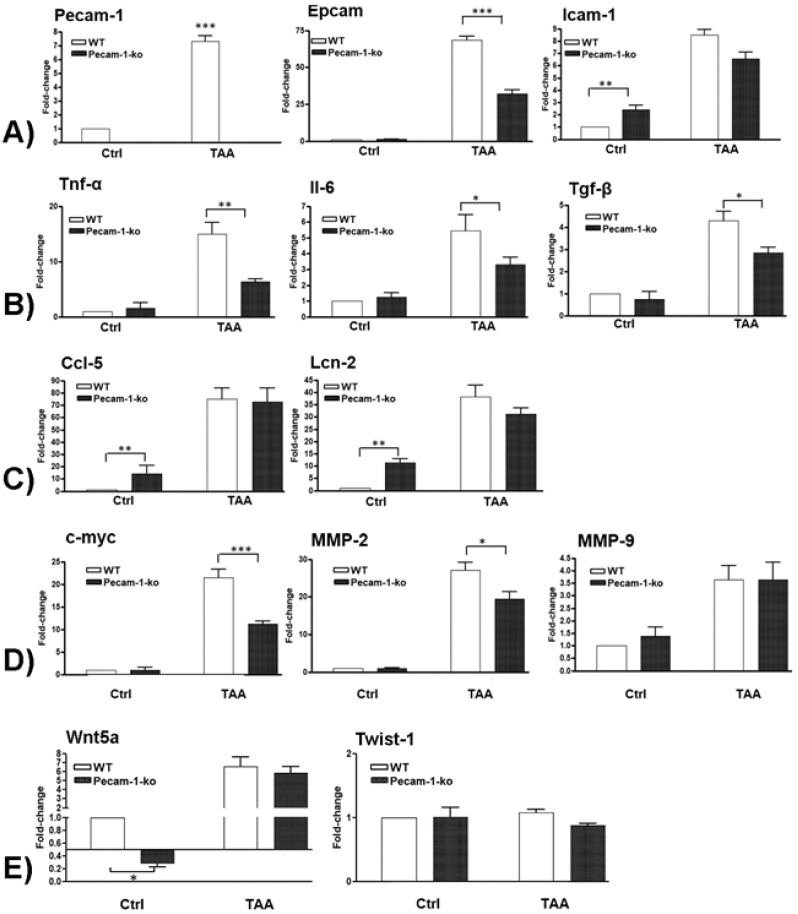
qRT-PCR analysis of total liver RNA from WT and Pecam-1 KO mice after 22 weeks of TAA in comparison to controls (Ctrl), which received only water. In all comparisons, the data were analyzed with WT control mice, which were set to 1. (**A**) Fold-change of mRNA expression of Pecam-1, Epcam and Icam-1. Note the significant upregulation of all three molecules after TAA, and significantly lower values of Epcam expression in TAA-treated Pecam-1 KO mice compared to WT mice. (**B**) Fold-change of mRNA expression of Tnf-α, Il-6 and Tgf-β. Note the significant upregulation after TAA, and significantly lower values in TAA-treated Pecam-1 KO mice compared to WT mice. (**C**) Fold-change of mRNA expression of Ccl5 and Lcn-2. Note the significant upregulation after TAA, with similar values in TAA-treated Pecam-1 KO mice vs. WT mice. Note the higher basic levels of Ccl-5 and Lcn-2 in Pecam-1 KO controls. (**D**) Fold-change of c-Myc, Mmp-2 and Mmp-9. Note the significant up-regulation of all three molecules after TAA, and significantly lower levels of c-Myc and Mmp-2 in Pecam-1 KO mice vs. WT mice. (**E**) Fold-change of Wnt5a and Twist-1. Note the significant up-regulation of Wnt5a, but not Twist-1, after TAA. Note significantly lower levels of Wnt5a in Pecam-1 KO control mice vs. WT-mice. For all: *n* = 6, measured in triplicates. Significance was accepted at * p ≤ 0.05, ** *p* ≤ 0.01, and *** *p* ≤ 0.001.

The changes in mRNA levels of important adhesion molecules were measured by qPCR in WT and Pecam-1 KO mice after 22 weeks of TAA treatment compared to controls. TAA administration induced a highly significant increase of the investigated adhesion molecules (Figure 9A). A significant induction (7.3 ± 0.4-fold) was observed in Pecam-1 gene expression after TAA administration in WT mice. Pecam-1 KO mice did not exhibit any Pecam-1 expression. The levels of Epcam were significantly up-regulated in both groups of mice after TAA administration compared to WT controls, however, the magnitude of induction was significantly higher in WT (68.5 ± 2.5-fold) compared to Pecam-1 KO mice (32.3 ± 2.5-fold). Icam-1 levels were higher in Pecam-1 KO control mice (2.4 ± 0.4-fold) as compared to WT mice basal. Compared to WT controls, Icam-1 levels increased significantly after TAA administration in both groups (WT= 8.5 ± 0.5 vs. KO= 6.6 ± 0.5-fold), but no significant difference between the WT and Pecam-1 KO group was observed (Figure 9A).

### 2.7. Changes in Hepatic Gene Expression of Pro-Inflammatory Cytokines

The changes in mRNA levels of major pro-inflammatory cytokines were measured by qPCR in TAA-treated WT and Pecam-1 KO-mice after 22 weeks compared to WT controls (Figure 9B). Significant up-regulation of all studied cytokines was observed, with increased Tnf-α levels in WT (14.9 ± 2.1-fold) and Pecam-1 KO mice (6.3 ± 0.6-fold). Similarly, an increase of Il-6 (WT = 5.5 ± 1-fold vs. Pecam-1-KO = 3.3 ± 0.4-fold) and Tgf-β was observed (WT = 4.3 ± 0.4-fold vs. Pecam-1 KO = 2.8 ± 0.3-fold). Notably, a significant difference in all of the studied cytokines was measured between TAA-treated WT and Pecam-1 KO mice, WT mice consistently showing higher levels (Figure 9B).

### 2.8. Changes in Hepatic Gene Expression of Ccl5 and Lcn-2

The changes in mRNA levels of inflammatory mediators Ccl5 and lipocalin-2 (Lcn-2) were measured by qPCR in TAA-treated WT and Pecam-1 KO mice after 22 weeks compared to WT controls. Thereby, the basal expression levels of Ccl5 (14 ± 7-fold) and Lcn-2 (11.5 ± 1.55-fold) were significantly higher in Pecam-1 KO control mice compared to WT mice (Figure 9C). After TAA administration, Ccl5 levels increased significantly in both WT mice (75 ± 9-fold) and Pecam-1 KO mice (72.6 ± 11.2-fold). A similar behavior was also observed for Lcn-2, with a significant increase after TAA in both WT mice (38 ± 5-fold) and Pecam-1 KO mice (31.3 ± 2.3-fold). However, there was no significant difference between the TAA-treated groups (Figure 9C).

### 2.9. Changes in Hepatic Gene Expression of c-Myc, Mmp-2, and Mmp9

Next, we measured the changes in mRNA levels of the pro-oncogenic molecules c-Myc, Mmp-2 and Mmp-9. Compared to controls, a significant up-regulation of c-Myc was observed after TAA administration both in WT (21 ± 1.8-fold) and Pecam-1 KO mice (11 ± 0.6-fold) (Figure 9D). A similar increase of Mmp-2 (WT = 27 ± 2; KO = 19.4 ± 1.9-fold) was detected. Both c-Myc and Mmp-2 showed a significantly higher expression in WT mice compared to Pecam-1 KO mice after TAAadministration. A significant upregulation of Mmp-9 mRNA was also observed after TAA administration in both groups of mice (WT = 3.6 ± 0.6; KO = 3.6 ± 0.7-fold), but there was no difference between the groups (Figure 9D).

### 2.10. Changes in Hepatic Gene Expression of EMT Markers

We then measured the changes in mRNA levels of Wnt5a and Twist-1 genes, which are known to be involved in the EMT process. A significantly reduced basal expression of Wnt5A mRNA was observed in Pecam-1 KO control mice (3 ± 0.5-fold) compared to WT controls, which received only water without TAA. Interestingly, a significant increase in gene expression of Wnt5a was observed after TAA administration both in WT (6.6 ± 1.1-fold) and Pecam-1 KO mice (5.8 ± 0.7-fold) (Figure 9E), compared to WT controls. No difference between the TAA groups was noticed. The expression of Twist-1 remained unchanged in all settings (Figure 9E).

## 3. Discussion

### 3.1. Animal Models of Intrahepatic Cholangiocellular Carcinoma

Intrahepatic cholangiocellular carcinoma (ICC) is a fatal cancer with only very limited therapeutic options. The etiology of ICC includes chronic inflammation, infection and sclerosis. The development of fibrosis and primary liver cancer is a multifaceted process that cannot be studied in humans. Reproducible animal models recapitulating the human disease are needed. Thereby, rodent models can be very helpful to unravel basic mechanisms, however, their translational value is often limited. For instance, the impact of heterotopic xenograft models in ICC is questionable [21], because ICC usually is a consequence of inflammation, and immunodeficient animals are not suitable for such studies. Bile duct ligation (BDL) or partial hepatic bile duct ligation are the best accepted models for cholestatic liver damage, but the rapid advancement to death of BDL animals limit its use for therapeutic interventions [27]. The model is useful to study acute cholestasis, but not chronic disease. Furthermore, genetically engineered mice (GEM) do not represent the bulk of genetically healthy patients [21], and there are only very few GEM models for ICC [28].

There is an utmost need for animal models which mimic human ICC. Previously, we could show the development of both HCC and ICC after 18 weeks of TAA administration in rats [29]. Based on our experience with rats, we sought to develop a murine hepatic carcinoma model by oral application of TAA. Tumor development occurred later than in rats, but after 22 weeks of TAA administration, 100% of the treated mice developed ICC. The 22 weeks were selected as an end point, after the mice were regularly sacrificed after 18 weeks to analyze the ICC progression. In mice, it obviously takes longer to induce ICC than in rats, indicating different metabolic capacities of the two rodents. If, additionally, there is development of HCC after prolonged TAA application is a matter of future experiments. Our anti-Ceacam-1 studies show that ICC in mice disrupts the bile canaliculi in hepatocytes, indicating cholestasis and hepatic stress, which may result in HCC after a prolonged period. This, however, remains to be studied.

The fact that ICC can be induced without any injections, but by application of the hepatotoxin in drinking water, this makes the model practical and highly reproducible. Like in the human disease, the model is characterized by the typical nodular appearance of the liver; accumulation of αSMA-positive connective tissue, diagnosed as stage-4 cirrhosis; and significant upregulation of liver serum markers ALT, AST, LDH, and bilirubin. ICC is clearly diagnosed by the abundance of Ck-19-positive biliary cells. The tumor cells form extended networks are mostly located in the interlobular septa. Additionally, dilated bile ducts are regularly seen as a sign of cholestasis. The large number of scattered Ck-19+ cells indicates EMT. This is also supported by the fact that many Ck-19 cells showed the morphology of ‘tip cells’ with filopodial extensions. The mechanisms of cholangiogenesis observed in our model seem to be highly similar to those in angiogenesis and lymphangiogenesis. In murine ICC, we observed upregulation of the non-canonical WNT-ligand Wnt5a. The importance of Wnt5a for angiogenesis and lymphangiogenesis has clearly been shown [30,31], and in other tumor entities, Wnt5a has been characterized as a motility-promoting factor [32]. Such functions may be preserved in ICC, and, in sum, the features described above characterize our mouse model as highly similar to the human ICC [33].

Our studies show upregulation of pro-inflammatory and pro-oncogenic molecules during ICC development. These include the adhesion molecules Icam-1 and Epcam, which are biomarkers for ICC [34,35]. Epcam has also been described as a biomarker for cancer stem cells. Accordingly, the levels of c-Myc, Mmp2 and Mmp9 greatly increased in ICC bearing mice. Notably, c-Myc can be expressed in biliary cells as well as in hepatocytes and inflammatory cells, and its induction has been positively linked with the progression of ICC [36,37]. Loss of the tight control of the activity of MMPs in the context of the tumor microenvironment increases the degradation of extracellular matrix (ECM) and can induce neovascularization, tumor spread and metastasis. Increased levels of Mmps in ICC correlate with tumor aggressiveness and metastasis [38,39].

### 3.2. Role of PECAM-1 in Inflammation-Induced Cancer

Pecam-1 has multifaceted pro- and anti-inflammatory functions in various settings [10], and we were interested in what the effects of the complete loss of Pecam-1 in liver cancer might be. We studied the functions of Pecam-1 in greater detail by inducing ICC in Pecam-1-null mice in parallel to WT mice. TAA administration induces the expression of Pecam-1 in the liver of WT mice, while Pecam-1-null mice remained negative. Our data show a disease-accelerating effect of Pecam-1, since the Pecam-1-null mice showed reduced inflammation, a lower stage of liver cirrhosis, and less ICC development.

PECAM-1 is an adhesion molecule, which mediates leukocyte trans-endothelial migration (TEM) [10,12]. PECAM-1 owns a cytoplasmic domain with two tyrosine residues. Tyrosine (Tyr)-690 is reported to be important not only for TEM but also for the transport of PECAM-1 towards and away from the lateral border recycling compartment (LRBC) of endothelial cells. Related molecules are also involved in TEM. Thereby, CD177 controls TEM of neutrophils via heterophilic interactions with PECAM-1 [40].

As mentioned above, the loss of Pecam-1 protected the mice to some degree from ICC progression. We observed less ICC-covered area in the liver and lower Ki-67 labelling of Ck-19-positive tumor cells. Also, compared to WT mice, lower levels of liver markers (AST, ALT, Bil), cytokines (Tnfα, Tgfβ, Il6), and tumor markers (c-Myc, Mmp2, Epcam) in Pecam-1-deficient mice after TAA-application underline the important functions of PECAM-1. It controls inflammatory processes by influencing the release of cytokines, leukocyte infiltration into inflammatory sites as well as maintenance and restoration of the vascular barrier integrity [41].

Additionally, it is worth noticing that PECAM-1 may have differential pro- and anti-inflammatory functions during acute and chronic settings. Such a biphasic role is supported by our findings in control mice. We observed significantly higher basal expression of Icam-1, Ccl-5 and Lcn-2 in Pecam-1-null mice, as well as higher numbers of neutrophils as compared to WT mice. Icam-1 facilitates the binding of leukocytes to vascular endothelium after extravasation, and CCL-5 is highly expressed by T-cells and can recruit leukocytes to the tumor microenvironment [42], indicating a pro-inflammatory function, which is increased in the absence of Pecam-1. In contrast, LCN-2 has a hepatoprotective function [43], which also increases in the absence of Pecam-1 and clearly shows bidirectional signaling of Pecam-1 under physiological conditions.

Moreover, we observed a larger number of neutrophils in the liver of untreated Pecam-1 KO mice compared to WT mice. Anti-tumor activity of activated N1 neutrophils by the release of pro-inflammatory mediators (e.g., chemokines) has been reported [44]. Indeed, acute inflammation expedites the anti-tumor response [8]. In the same line, it is conceivable that higher neutrophil numbers and elevated levels of inflammatory mediators in Pecam-1-null mice delay the progression of cancer. Further support for this hypothesis comes from the specific localization of neutrophils adjacent to the Ck-19+ cells in our ICC model. In physiological conditions, only few neutrophils are scattered throughout the liver, and increased numbers come in close contact with biliary cells in diseased stages. Their distinction into specific anti- and pro-tumorous populations need to be studied in greater detail.

Chronic inflammation has often been associated with cancer development. However, it is still not clear how chronic inflammation initiates cancer [5,8]. Of note, inflammatory cells release metabolites such as reactive oxygen species (ROS), which can induce DNA damage of neighboring cells, e.g., biliary cells in our model. Recently, PECAM-1-low angiosarcoma cells have been shown to be more efficient in ROS detoxification than PECAM-1-high cells [45]. It is likely that Pecam-1-null mice in our model may better overcome TAA-induced ROS than WT mice. This may explain less inflammation and tumor progression in Pecam-1 mice.

PECAM-1 has also been implicated in fibrosis, spreading of primary tumors, control of proliferation, vascular permeability, and capillary morphogenesis [14,46]. PECAM-1 is a marker for endothelial cells and its expression has been found in several types of human cancers [16,17,18,20]. Anti-PECAM-1 therapy was shown to inhibit the progression of already developed tumor metastases by reducing proliferation [14]. The reduction of PECAM-1 expression by siRNA was shown to be linked to the inhibition of fibrogenesis and metastasis in the lung [47].

Taken together, we present the first highly reproducible mouse model for ICC and show protective effects of Pecam-1 deficiency.

## 4. Material and Methods

### 4.1. Materials

If not mentioned otherwise, all chemicals and reagents were purchased from Sigma-Aldrich (St. Louis, MO, USA) and Merck (Darmstadt, Germany).

### 4.2. Animals

C57BL/6 and Pecam-1 knock out (KO) mice (B6.129S-*Pecam1^Gt(OST16303)Lex^*/J) were purchased from The Jackson Laboratory (Bar Harbor, ME, USA). All mice used in this study were male, 6–8 weeks-old, and had a body weight of 20–28 g. All mice received food and drinking water ad libitum. The mice were divided into four groups. Groups 1 and 2, which contained WT and homozygous Pecam-1 KO mice, respectively, received pure drinking water. In group 3, 4500 mg/L thioacetamide (TAA; Merck, Darmstadt, Germany) was added to the drinking water of WT and KO mice. After 22 weeks, all mice were sacrificed. Liver and blood were used for subsequent analyses. The animal studies were reviewed and approved by the committee of the Central Institute for Animal Experiments of the University Medical Center Goettingen, UMG, and the Lower Saxony State Office for Consumer Protection and Food Safety (LAVES study No. 33.9-42502-04-15/1806).

### 4.3. Quantification of Hepatic Markers in Murine Serum

Blood samples were collected from the *V. cava inferior* from all groups of mice. The collected serum was used for alanine aminotransferase (ALT), aspartate aminotransferase (AST), lactate dehydrogenase (LDH), and bilirubin (Bil) measurements in collaboration with the routine diagnostic service of the University Medical Center Goettingen, UMG, Germany.

### 4.4. Immunofluorescence of Murine Liver Sections

Immunofluorescence staining was executed on 5-µm-thick cryosections of the liver as described [41]. The cold acetone/methanol fixed sections were washed with PBS. Afterwards, the sections were treated with blocking solution (0.1% BSA, 10% FCS in PBS) for 1h, and further incubated with primary antibody at 4 °C overnight.

Primary antibodies are listed below. Non-immune serum was used as a negative control. Secondary antibodies which were bought from Molecular Probes (Leiden, The Netherlands) are also listed below. Nuclear counterstaining was performed by DAPI (4′,6-diamidino-2-phenylindole; Southern Biotech, Birmingham, AL, USA). The stained sections were studied with AxioImager Z1 epifluorescence microscope (Zeiss, Jena, Germany). Antibodies used in the study are described in Table 2.

The quantification of Ck-19 was performed by measuring the Ck-19-positive fluorescent area, obtained from the liver sections stained with Ck-19 antibody, through ImageJ Software [48]. The Ki-67-positive Ck-19 cells were quantified by counting manually the double positive fluorescent cells stained in liver sections using antibodies against Ki-67 and Ck-19. A similar method was used for the quantification of Gr-1-postive cells using antibody against Gr-1.

### 4.5. RNA Isolation and Real-Time PCR Analysis

Total RNA was extracted from the livers of Pecam-1 KO and WT mice after homogenization in Trizol (Invitrogen, Carlsbad, CA, USA). q-RT-PCR was carried out as described before [34,35] with primers purchased from IBA Lifesciences (Goettingen, Germany). Sequences of the primers are as follow:

*Pecam-1*: forward, AACAGAAACCCGTGGAGATG, reverse, GTCTCTGTGGCTCTCGTTCC;
*Icam-1*: forward, ATTCGTTTCCGGAGAGTGTG, reverse, CAGCACCGTGAATGTGATCT;
*EpCAM*: forward, GCTGTCATTGTGGTGGTGTC, reverse, TGGATCTCACCCATCTCCTT;
*Tgf-β*: forward, TTGCTTCAAGCTCCACAGAGA, reverse, CAGAAGTTGGCATGGTAGCC;
*Tnf-α*: forward, CAAACCACCAAGTGGAGGAG, reverse, GTGGGTGAGGAGCACGTAGT;
*Il-6*: forward, CAAAGCCAGAGTCCTTCAGAG, reverse, GAGCATTGGAAATTGGGGTA;
*Ccl5/Lix*: forward, GGTCCACAGTGCCCTACG, reverse, GCGAGTGCATTCCGCTTA;
*Lcn-2*: forward, AAATTGCACAGGTATCCTCAG, reverse, CAGAGAAGATGATGTTGTCGT;
*C-Myc*: forward, CGGACACACAACGTCTTGGAA, reverse, AGGATGTAGGCGGTGGCTTTT;
*Mmp-2*: forward, GCTGTATTCCCGACCGTTG, reverse, TGGTCCGCGTAAAGTATGGG;
*Mmp-9*: forward, ACGACATAGACGGCATCCAGTATC, reverse, AGGTATAGTGGCACACATAGTGGG;
*Wnt-5a*: forward, AAGCAGGCCGTAGGACAGTA, reverse, CAGCACGTCTTGAGGCTACA;
*Twist-1*: forward, GAGCAAGATTCAGACCCTCAA, reverse, CATCTTGGAGTCCAGCTCGT;
*B2m*: forward, ATTCACCCCCACTGAGACTG, reverse, ATCCCAGTAGACGGTCTTGG;
*GAPDH*: forward, AGAACATCATCCCTGCATCC, reverse, CACATTGGGGGTAGGAACAC.

### 4.6. Statistical Analysis

The data were analyzed using Graph pad Prism version 4 software (San Diego, CA, USA). All experimental errors are shown as SEM. Statistical significance was calculated with Student’s t-test. Significance was accepted at * p ≤ 0.05, ** *p* ≤ 0.01, and *** *p* ≤ 0.001.

### 4.7. Data Availability

All data are included in the manuscript.

## 5. Conclusions

We establish the first mouse model of intrahepatic cholangiocellular carcinoma (ICC) in wild-type and Pecam-1-null mice using thioacetamide in drinking water. After 22 weeks, cirrhosis and ICC are present, but are less severe in Pecam-1-null mice. ICC is mainly located in portal areas and resembles the human ICC. We present a highly reproducible mouse model for ICC, as prerequisite for pre-clinical drug testing. Pecam-1-related signaling may be an attractive target to inhibit liver-disease progression.

## Figures and Tables

**Table 1:** Histological staging and inflammation grade of livers stained with HE and trichrome after TAA administration in WT and Pecam-1 KO mice.

Mouse	Liver Disease	Stage	Inflammation Grade
WT	Cirrhosis	4	1–2
WT	Cirrhosis	4	1–2
WT	Cirrhosis	4	1–2
WT	Cirrhosis	4	1–2
WT	Cirrhosis	4	1–2
WT	Cirrhosis	4	1–2
Pecam-1-KO	Cirrhosis	3	1
Pecam-1-KO	Cirrhosis	3	1
Pecam-1-KO	Cirrhosis	3	1
Pecam-1-KO	Cirrhosis	3	1
Pecam-1-KO	Cirrhosis	3	1
Pecam-1-KO	Cirrhosis	3	2

**Table 2:** Antibodies used in the study.

Antibody	Company	Cat. Number	IHC
rb-anti-ms-CK-19	Abcam	ab 76539	0.180556
rt-anti-F4/80 (BM8)	Abcam	ab 16911	1:10
rt-anti-ms Ly-6G/ly-6C (Gr-1)	Invitrogen	14-5931-81	0.180556
sh-anti-ms-CEACAM-1	R &D systems	AF-6480	0.111111
ms-anti-SMA	Sigma	A-2547	0.180556
**Secondary Antibodies:**			
gt-anti-rt			
gt-anti-rb	Molecular Probes	---	0.180556
dk-anti-rb			
dk-anti-sh	Dianova	713-225-147	0.180556
